# Obituary

**Published:** 1917-11-24

**Authors:** 


### Obituary.

We regret to record the sudden death on the 14th inst. of Mr. J. W. Cross, pharmacist and dispenser to the Leicester Royal Infirmary, at the age of forty-one. Mr. Cross, after feeling indisposed for a few days, was taken seriously ill on Saturday, the 10th instant, and admitted to the infirmary in a semi-conscious condition, with symptoms of acute pneumococcic meningitis, to which he succumbed.

A correspondent writes: "His work at the infirmary was marked by great devotion and singleness of aim. He loved his work, he loved the institution; he was never happier than when giving helpful advice to the large staff with whom he worked, and by whom he was held in the highest possible esteem. No man worked harder for his institution than he, and it is typical and characteristic of the man that his books and papers are left in order and completeness, so that his methods have only to be continued to secure the same perfect working system which has always characterised his department. He leaves behind a memory which will long be recalled with gratitude for many acts of kindness, and an example which will inspire in others a sense of devotion and duty."

